# Compensation or inhibitory failure? Testing hypotheses of age-related right frontal lobe involvement in verbal memory ability using structural and diffusion MRI

**DOI:** 10.1016/j.cortex.2014.08.001

**Published:** 2015-02

**Authors:** Simon R. Cox, Mark E. Bastin, Karen J. Ferguson, Mike Allerhand, Natalie A. Royle, Susanna Muñoz Maniega, John M. Starr, Alasdair M.J. MacLullich, Joanna M. Wardlaw, Ian J. Deary, Sarah E. MacPherson

**Affiliations:** a Brain Research Imaging Centre, University of Edinburgh, Edinburgh, UK; b Centre for Cognitive Ageing and Cognitive Epidemiology, University of Edinburgh, Edinburgh, UK; c Department of Psychology, University of Edinburgh, Edinburgh, UK; d Scottish Imaging Network: A Platform for Scientific Excellence (SINAPSE) Collaboration, UK; e Geriatric Medicine, University of Edinburgh, Edinburgh, UK; f Alzheimer Scotland Dementia Research Centre, University of Edinburgh, Edinburgh, UK; g Endocrinology Unit, University of Edinburgh, Edinburgh, UK

**Keywords:** Verbal memory, Cognitive ageing, Compensation, MRI, Frontal lobe, Corpus callosum

## Abstract

Functional neuroimaging studies report increased right prefrontal cortex (PFC) involvement during verbal memory tasks amongst low-scoring older individuals, compared to younger controls and their higher-scoring contemporaries. Some propose that this reflects inefficient use of neural resources through failure of the left PFC to inhibit non-task-related right PFC activity, via the anterior corpus callosum (CC). For others, it indicates partial compensation – that is, the right PFC cannot completely supplement the failing neural network, but contributes positively to performance. We propose that combining structural and diffusion brain MRI can be used to test predictions from these theories which have arisen from fMRI studies. We test these hypotheses in immediate and delayed verbal memory ability amongst 90 healthy older adults of mean age 73 years. Right hippocampus and left dorsolateral prefrontal cortex (DLPFC) volumes, and fractional anisotropy (FA) in the splenium made unique contributions to verbal memory ability in the whole group. There was no significant effect of anterior callosal white matter integrity on performance. Rather, segmented linear regression indicated that right DLPFC volume was a significantly stronger positive predictor of verbal memory for lower-scorers than higher-scorers, supporting a compensatory explanation for the differential involvement of the right frontal lobe in verbal memory tasks in older age.

## 1 Introduction

Some aspects of memory functioning decline with age (Craik & Rose, 2012). Functional imaging studies of verbal memory tasks that compare activation patterns between young and older people show that older age is accompanied by “over-recruitment”; that is, greater cortical activation both in the brain regions engaged by young subjects, and also in a more distributed network that has additional regions (reviewed in Craik & Rose, 2012; Goh, 2011; Park & Reuter-Lorenz, 2009). What this over-recruitment might represent is a matter of debate. Some authors have posited that it reflects an attempt to supplement the functioning of a failing network and thus makes a positive compensatory contribution to memory performance (Cabeza, Anderson, Locantore, & McIntosh, 2002; Park & Reuter-Lorenz, 2009). Others propose that such differences could reflect changes that are potentially detrimental to cognitive performance, either through general breakdown in the functional specialization of the cortex (Li, Brehmer, Shing, Werkle-Bergner, & Lindenberger, 2006) or an inability to shut down activity not related to the cognitive task being performed (Logan, Sanders, Snyder, Morris, & Buckner, 2002). However, a breakdown in functional specialisation could also be compatible with a compensatory interpretation of over-recruitment, and as such these cannot be treated as mutually exclusive accounts. In the current study, we propose that the use of structural MRI data can provide an alternative perspective for testing hypotheses on this phenomenon that have arisen from the functional neuroimaging literature.

One brain region that has been shown to exhibit age-related over-recruitment during verbal memory encoding is the right prefrontal cortex (PFC). Activation of the right PFC has been reported in older, but not younger participants, in addition to the expected blood oxygen level dependent (BOLD) response found in the left lateral PFC and bilateral medial temporal lobe in young participants during verbal memory recall tasks (de Chastelaine, Wang, Minton, Muftuler, & Rugg, 2011; Duverne, Motamedinia, & Rugg, 2009; Logan et al., 2002; Morcom & Friston, 2012; Morcom, Good, Frackowiak, & Rugg, 2003; Reuter-Lorenz et al., 2000). Moreover, these additional rightward-frontal activations are not necessarily present in every individual within the older group, but are associated with poorer memory performance (de Chastelaine et al., 2011; Duverne et al., 2009; Persson et al., 2006). In other words, the older individuals who tend to perform more poorly on memory encoding tasks tend also to be the members of their age group who exhibit the greatest additional right PFC activity.

This link between *increased* right frontal BOLD activity and *poorer* memory performance is intuitively more consistent with an inability to direct neural resources to the task being performed than with the view that right PFC makes positive contributions to performance. Some authors have argued that, during verbal memory tasks which are usually supported by strongly lateralised neural activity, reduced callosal integrity facilitates coactivation of homotopic cortex that is detrimental to performance (Buckner & Logan, 2002; Logan et al., 2002). Thus, increased BOLD activity in poorer performers might arise due to an inability of the left frontal lobe to inhibit the right via the anterior corpus callosum (CC). Presumably then, lower volume of the left PFC and integrity of the CC leads to impaired trans-callosal inhibition and additional recruitment of the right PFC found in functional MRI (fMRI) studies. We shall refer to this as the *inhibitory hypothesis*. However, another possible interpretation is that of partial compensation (Duverne et al., 2009; Rossi et al., 2004), which suggests that whatever auxiliary processing is facilitated by the additional activation found in some older people is not sufficient to fully replicate a normally-functioning network, but would lead to much poorer performance if this alternative cognitive route were not available. We shall refer to this as the *partial compensation hypothesis*.

Predictions from these two hypotheses can be formalised and usefully tested by examining the neurostructural correlates of verbal memory performance in older age. We address this question from the following viewpoint: Disruption to one or more components of the large-scale brain network involved in memory may disrupt the state of normal parallel processing necessary to support unhindered performance (Bressler & Menon, 2010; Mesulam, 1990). Accumulated brain insults over the life course may well be such a mechanism of disruption. For each component of the large-scale memory network, such insults can be broadly indexed by individual differences in diffusion and structural MRI measures (white matter tract integrity parameters and regional brain volumes controlled for intracranial volume). We shall therefore use structural brain measures of an *a priori* selection of memory network components (hippocampus, CC and lateral frontal lobe) to test competing accounts of frontal lobe involvement in verbal memory performance among a group of healthy older adults in their early 70s.

We first aim to verify that left frontal lobe, hippocampus and CC constitute parts of a memory network and that each contributes unique variance to memory performance (Bressler & Menon, 2010; Mesulam, 1990). The inhibitory hypothesis would predict positive associations between memory ability and indices of left lateral frontal lobe and anterior CC (genu; Buckner & Logan, 2002; Logan et al., 2002; Persson et al., 2006; Sullivan & Pfefferbaum, 2007). Furthermore, a significant positive relationship between right frontal volume and memory ability would be incompatible with the inhibitory hypothesis, which suggests no benefit to verbal memory performance from a larger right frontal lobe. Conversely, the partial compensation hypothesis (Duverne et al., 2009; Rossi et al., 2004) would assert that larger volume of the area providing auxiliary processing (in this case, the right frontal lobe) would positively associate with memory score, but only for poorer performers, who putatively rely on its compensatory function. Thus, the association between right frontal lobe volume and verbal memory score should be 1) significantly positive in lower, but of no significant benefit to higher performers, and, 2) of a significantly different magnitude between lower and higher performers.

However, using structural MRI variables and cognitive scores does not allow us to parse apart the contributions that brain regions might differentially make to encoding and retrieval phases of a memory task (an undeniable advantage of fMRI). The right frontal lobe has been implicated in monitoring/checking processes during retrieval of some types of information (Cabeza, Locantore, & Anderson, 2003; Fletcher, Shallice, Frith, Frackowiak, & Dolan, 1998; Henson, Shallice, & Dolan, 1999). One might therefore argue that any associations between cognitive score and right frontal lobe volume cannot be ascribed to compensatory encoding (for example) to the exclusion of retrieval processes. Nevertheless, the data on frontal lateralisation of retrieval processes is far from clear-cut (Fletcher & Henson, 2001) and some studies have implicated the right frontal lobe only in retrieval of non-verbal material and the left frontal lobe in retrieval of verbal material (Fletcher et al., 1998; McDermott, Buckner, Peterson, Kelley, & Sanders, 1999; Opitz, Mecklinger, & Friederici, 2000; Wagner et al., 1998), whereas others suggest that only less demanding tasks are more likely to show right lateralised prefrontal activation (reviewed in Nolde, Johnson, & Raye, 1998) or that task requirements (recall *vs* recognition) are key (Cabeza et al., 2003). A recent meta-analysis of 30 studies identified a predominantly left frontal BOLD response associated with retrieval success, though this was based on old-new recognition paradigms rather than free or cued recall as used in the present study (Spaniol et al., 2009). Notwithstanding the lack of clarity regarding right frontal involvement in verbal memory retrieval, such a role would become apparent in a group-wide positive association between right frontal volume and memory performance in the current study. This provides a clear contrast to the predictions set out by the compensatory hypothesis (differential associations based on performance), and would have no bearing on the inhibitory hypothesis which concerns the left frontal lobe and anterior CC.

## 2 Materials and methods

### 2.1 Participants

Study participants comprise a subset of 90 males from the Lothian Birth Cohort 1936 (LBC1936). The members of this cohort were born in 1936 and most sat a well-validated general mental ability (IQ-type) test at school in Scotland in 1947 at an average age of 11 years. At around 70 years of age, 1091 surviving, healthy, community-dwelling residents in the Edinburgh area who had taken this initial test were recruited as the LBC1936. The initial wave of testing contained this same mental test in addition to other cognitive and medical tests which are detailed elsewhere (Deary et al., 2007). Three years later, 866 returned for a second follow-up wave of cognitive testing and an MRI brain scan (Deary, Gow, Pattie & Starr, 2012; Wardlaw et al., 2011). From this second wave, the participants for the present study were selected on the following criteria: a score of 24 or greater on the Mini-Mental State Exam (MMSE; Cockrell & Folstein, 1988), a score of less than 11 on the depression facet of the Hospital Anxiety and Depression Scale (Snaith, 2003), not taking any antidepressant or glucocorticoid medication, and inclusion in a glucocorticoid study for which participants were all male. Participants reported no serious neurodegenerative diseases at interview, nor exhibited clinically significant cerebral features on MRI as assessed by a consultant neuroradiologist (JMW).

Written informed consent was obtained from each participant prior to testing, which was conducted in compliance with departmental guidelines on participant testing and the Declaration of Helsinki. Ethical approval was gained from NHS Lothian Research Ethics Committee and the Philosophy, Psychology and Language Sciences Research Ethics Committee at the University of Edinburgh.

### 2.2 Cognitive test scores

Immediate verbal memory was assessed using Logical Memory (LM) and Verbal Paired Associates (VPA) tests from the Wechsler Memory Scale III^UK^ (WMS-III; Wechsler, 1998). In LM part I, participants are presented with two stories that both contain 25 elements. The first story is read aloud, and then scored based on the number of elements recalled by the participant immediately after reading. The second story repeats this pattern twice, and the participant is informed they will be tested again later. In LM part II, following an approximately 30 min delay, scores are based on the ability to recall as many items as possible from the two stories. For the VPA part I, eight pairs of unrelated words are read to participants. Without a delay, they are then given the first item of each pair and ask to recall the associated word. This procedure using the same 8 word pairs is repeated a further three times with no delay. In VPA II, there is one further trial following a 30 min delay, in which the word pairs are not read out first. Immediate verbal memory recall was assessed using the LM I and VPA I scores and delayed verbal memory recall was assessed using LM II and the VPA II scores. These tests exhibit good test-retest reliability in participants aged 70–74 years; LM I = .81, LM II = .77, VPA I = .94 and VPA II = .87 (Wechsler, 1997).

### 2.3 MRI acquisition

Full details of the brain MRI protocol, including figures illustrating the images acquired, are available in Wardlaw et al. (2011). Briefly, participants were scanned using a GE Signa Horizon HDxt 1.5 T clinical scanner (General Electric, Milwaukee, USA). Image acquisition took approximately 70 min, and comprised whole brain T2-, T2*- and FLAIR-weighted axial scans, a high-resolution 3D T1-weighted volume sequence acquired in the coronal plane (voxel dimensions 1 × 1 × 1.3 mm), and a diffusion MRI protocol consisting of seven T2-and sets of diffusion-weighted (*b* = 1000 sec/mm^2^) axial single-shot spin-echo echo-planar volumes acquired with diffusion gradients applied in 64 non-collinear directions (voxel dimensions 2 × 2 × 2 mm).

### 2.4 Sub-regional volumes

Volumes of the dorsolateral prefrontal cortex (DLPFC), inferior frontal gyrus (IFG) and the hippocampus in each hemisphere were derived from the T1-weighted scans and are reported in mm^3^. All volumetric analyses were performed blind to participant identity. The cytoarchitectural justification and landmarks used for the frontal volumetric measures have been published in detail elsewhere (Cox et al., 2014). Briefly, the frontal lobe regions-of-interest (ROIs) were manually delineated on consecutive coronal slices at 1.3 mm thickness on AC-PC aligned T1-weighted volume scans by one of the authors (SRC). Key landmarks were identified on each slice and boundaries drawn by connecting those sulci with straight lines. The DLPFC was ventrally limited by the inferior frontal sulcus, and medially by the crown of the most medio-superior gyrus. Both DLPFC and IFG were limited anteriorly by the frontal pole (a coronal plane at the most anterior extent of the cingulate sulcus or paracingulate sulcus where present), and posteriorly by a coronal plane at the most anterior extent of the precentral gyrus. The IFG was limited dorsally by the inferior frontal sulcus, and ventrally by the lateral orbital sulcus in more anterior slices, or the circular sulcus of the insula in more posterior slices. As such, the Brodmann areas (BA) broadly represented were BA46/9 (DLPFC) and BAs 44, 45 and 47 (IFG). Intra-class correlation coefficients (agreement; Shrout & Fleiss, 1979) and Bland-Altman analysis (Bland & Altman, 1986) were conducted based on the absolute regional volumes of 20 hemispheres, parcellated 2 weeks apart by the same rater (SRC) for IFG (ICC = .96, Bland Altman mean = .93, 95% C.I. = −11.81 to 13.67), and DLPFC (ICC = .99, Bland Altman mean = 1.19, 95% C.I. = −5.16 to 7.54).

The hippocampus was initially segmented automatically and then each output was manually edited. Initial automatic segmentation was conducted using FSL FIRST, in which the T1-weighted volume was registered to an age-appropriate template (Farrell et al., 2009) and then to an optimised sub-cortical mask. Visual assessment and manual editing of the object masks was then conducted by one of the authors (NAR) with an intra-rater correlation co-efficient of .98. Movement artefact in the anterior portion of two MRIs prevented prefrontal volumetric analysis, leaving 88 T1-weighted scans for the frontal sub-regions. Automated segmentation of the hippocampi failed in one case, leaving 89 participants with manually-edited hippocampal volumes.

### 2.5 Tractography

After pre-processing the diffusion MRI data to extract the brain, and remove bulk patient motion and eddy current induced artefacts, mean diffusivity (MD) and fractional anisotropy (FA) parametric maps were generated for every subject using tools freely available in FSL (FMRIB, Oxford, UK; http://www.fmrib.ox.ac.uk). Tract-averaged MD and FA values, weighted by the connection probability, were determined for genu and splenium of CC using probabilistic neighbourhood tractography with the BedpostX/ProbTrackX algorithm (Behrens, Berg, Jbabdi, Rushworth, & Woolrich, 2007), a novel approach for automatic and reproducible segmentation of major white matter pathways (Clayden et al., 2011; http://www.tractor-mri.org.uk).

### 2.6 Statistical analysis

Independent sample *t*-tests indicated that the 90 participants in the current study did not differ significantly from the other participants that attended wave 2 of LBC1936 testing for LM1 [*t* (862) = −1.15, *p* = .25], LM2 [*t* (862) = −1.31, *p* = .19], VPAI [*t* (843) = −1.20, *p* = .23] and VPAII [*t* (841) = −1.40, *p* = .16]. Pearson's correlations with large effect sizes between tests for scores of immediate [LM1 and VPA1; *r* (87) = .56, *p* < .001] and delayed recall [LMII and VPAII; *r* (87) = .50, *p* < .001] suggested that the test scores could be combined into two overall measures. Z-scores were created and averaged to yield two scores of verbal memory ability for each participant; one of Immediate (*M* = −.01, *SD* = .90) and one of Delayed recall ability (*M =* −.01, *SD* = .89). One participant did not complete the VPA, and so the score for LM performance was used in place of an average verbal memory ability score. Correlations among raw memory scores are given in Supplementary Table I.

All regional volumes were controlled for intracranial volume (ICV; reflecting maximal healthy brain size; Royle et al., 2013). As such, residuals derived from the linear regression between ICV and regional volume allow us to compare volumes across individuals, accounting for how large one would expect them to be given their maximal healthy brain size. Thus, two individuals with the same raw IFG volume (for example) are not necessarily treated the same; rather, the corrected value represents its actual size relative to its expected size within the sample. Though this is an imperfect measure that cannot take account of individual differences in the degree of tissue-specific change (for which longitudinal data are required), we contend that – particularly in the context of older participants – this step is preferable to using raw values, which cannot differentiate at all between participants with different levels of global atrophy. The resultant unstandardized residuals were used in all further analysis. Outlier (±3 SD) and normality checks were performed on all variables. The object maps of the outlying values were inspected (without knowledge of their relation to other variables) to check for measurement error. A single marginal outlier was identified in both left and right hippocampi, and they were winsorized following examination of object maps by one of the authors (NAR) in order to preserve data points but minimize the disproportionate effect of outlying points on parametric analyses. Tract segmentation quality was examined by one of the authors (SMM). Tracts affected by partial volume averaging of CSF signal or which contained aberrant or truncated pathways that were not anatomically plausible representations of the fasciculi-of-interest were excluded from further analysis, leaving 83 participants with genu and 86 with splenium integrity measures.

Initially, the relationships between measures of brain structure (FA and MD of the splenium and genu, and ICV-controlled volumes of the hippocampus, DLPFC and IFG) and memory performance (Immediate and Delayed verbal memory mean z-scores) were ascertained for the whole group. Hierarchical linear regression was used to examine the contribution each region made to the variance in memory performance (mean z-scores of Immediate and Delayed verbal memory). Cerebral volumes and diffusion parameters were entered step-wise into the model based on correlation magnitude (largest first) to establish whether each region contributed uniquely to variance in test score, consistent with concepts of large-scale brain networks (Bressler & Menon, 2010; Mesulam, 1990). Predictions based on the inhibitory hypothesis were also addressed with this analysis: that there would be a significant relationship between memory score and measures of both left frontal lobe and genu.

In the second part of the analysis, we addressed the partial compensation hypothesis in two parts: i) that right frontal volume is particularly beneficial to lower rather than higher performers and ii) that this is related to the poorer status of other memory network components. However, we opted not to split participants by their median z-scores into high and low groups – as has previously been the case in the preceding fMRI studies. Arbitrarily dichotomising a continuous variable such as memory score can be inappropriate as it might turn a normally-distributed set of data into two independent samples and result in erroneous classification of participants (MacCallum, Zhang, Preacher, & Rucker, 2002; Preacher, Rucker, MacCallum, & Nicewander, 2005). This is a particularly important point for cross-study comparison of high and low performers because – when split *a priori* – the dichotomy is based solely on the *relative* cognitive characteristics within that sample, rather than on the presence/absence of the actual phenomenon of interest (right frontal involvement, in this case). As a consequence, it cannot be assumed that each study recruits precisely 50% of participants who exhibit right frontal involvement during verbal memory tasks. This makes the comparison of reported characteristics for ‘low-performers’ between studies (and the practice of dichotomising on a median split of memory scores) potentially unreliable for investigating right frontal lobe involvement in memory ability. To this end, we used segmented linear modelling in the whole sample to test hypotheses outlined above for Immediate and Delayed recall. Segmented linear regression allows two different segments to be fitted within an overall group analysis, separated by a breakpoint. This allows us to empirically identify if – and where – there is a significant difference in the predictive value of a target ROI on memory score. Crucially, the groups are therefore not split by an arbitrary criterion (i.e., median memory z-score). Instead, we used a function to empirically search for any potential breakpoints where the slopes of the two segments are significantly different, according to memory score.

Thus, we fitted a two-segment model parameterized so as to estimate the difference in linear slope between the segments. The model was fitted using 120 breakpoints in order to locate the memory scores at which there was a significant (*p* < .05) difference between segment slopes. The significant breakpoint that divided group size most evenly (in order to distribute power between segments as equally as possible) was then identified, and the model was then re-parameterized to estimate and test the slopes of the two segments joined at this breakpoint. This was conducted for right frontal volumes (DLPFC and IFG) with Immediate and Delayed recall score.

We then created a general measure of memory network integrity for each participant. We created standardised scores (mean = 100, SD = 15) for each MRI variable significantly associated with memory at the group level, and then compared the means between the participants on either side of the breakpoint. The compensatory hypothesis would predict that poorer performers would have a significantly lower mean score than their counterparts.

Our sample included 8 left-handed participants. It has been proposed that the role of handedness may be particularly relevant to performance on some verbal memory tasks, such as paired associate recall (e.g., Lyle, McCabe, & Roediger, 2008). As such, we conclude by conducting sensitivity analysis, to check for any confounding of handedness on the reported results.

## 3 Results

**Fig. 1:**
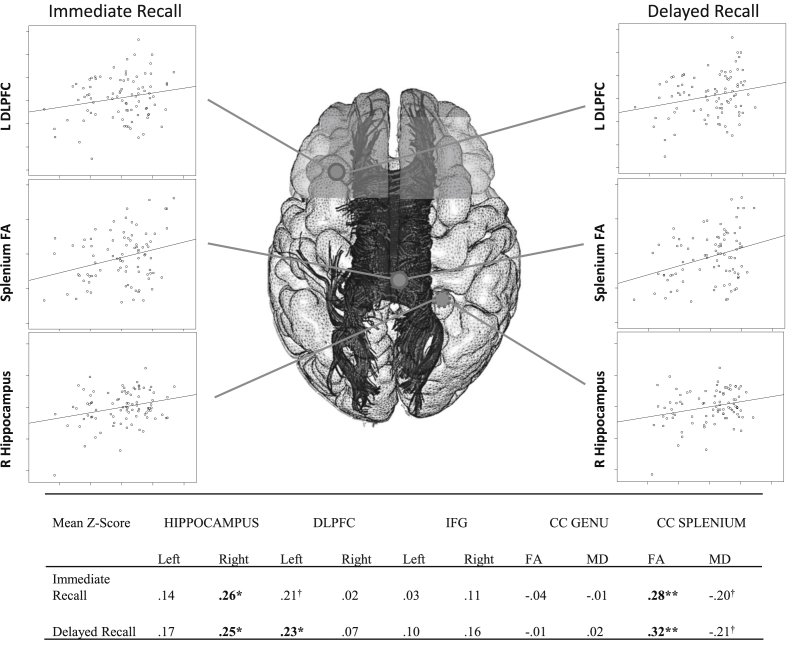
Correlations between brain structure and mean z-scores for Immediate and Delayed verbal memory recall for the whole group (*x*-axis runs from z = −2 to 2 in increments of 1). ^†^trend (.05 < *p* < .08), *p < .05, **p < .01; DLPFC: dorsolateral prefrontal cortex; IFG: inferior frontal gyrus, FA: fractional anisotropy (*y*-axis runs from FA = .3 to .7 in increments of .1), MD: mean diffusivity. Hippocampus (*y*-axis runs from −1000 to 1000 in increments of 500), IFG and DLPFC (*y*-axis runs from −15,000 to 15,000 in increments of 5000) are volumes (mm^3^) controlled for intracranial volume. Figure amended with permission from Catani & Thiebaut de Schotten, 2008).

Participant characteristics are described in Table 1 and the correlations among brain imaging variables can be found in Supplementary Table II. Compared to normed data for 70–74 year olds (Wechsler, 1998), participants' mean scores on subtests were within the normal range, but slightly above the average scaled score of 10 on LM (scaled score = 13 for both I and II) and VPA (part I scaled score = 12, part II scaled score = 13). Within this, scaled scores ranged from very high to very low scores on LM (scaled score of 3–18 for part 1 and 4–19 for part II) and VPA (5–18 for part 1, 5–15 to II). Frontal volumes were generally well-correlated (*r* > .26, *p* < .05) apart from a non-significant correlation between right IFG and left DLPFC. Frontal volumes did not correlate significantly with callosal measures, nor were splenium and genu measures significantly related. We conducted correlations between the two verbal memory indices (Immediate and Delayed) against the 10 MRI-derived measures (bilateral region volumes of the IFG, DLPFC, and hippocampus, and FA and MD of the callosal splenium and genu) (Fig. 1). Participants' scores on Immediate and Delayed verbal memory recall were significantly positively correlated with the volume of the right hippocampus, left DLPFC and splenium FA (magnitudes .23 to .32, *p* < .05); there was a trend towards significance for the relationship between left DLPFC volume and Immediate verbal memory recall. These relationships were significantly lateralised to left DLPFC for Immediate recall [*t* (85) = 2.02, *p* = .046] and a trend for Delayed [*t* (85) = 1.70, *p* = .093], but not to the right for the hippocampus [Immediate: *t* (86) = 1.24, *p* = .218; Delayed: *t* (86) = .83, *p* = .411]. The magnitude of the effect was significantly greater in the splenium than the genu in terms of FA [Immediate: *t* (79) = 2.23, *p* = .028; Delayed: *t* (79) = 2.31, *p* = .023] but not MD [Immediate: *t* (79) = 1.29, *p* = .202; Delayed: *t* (79) = 1.51, *p* = .136].

When entered into a linear regression, variability in these regions predicted 16% of the variance in Immediate and 19% in Delayed verbal memory recall for the overall sample (Table 2). Each step-wise iteration showed an increase in *R*^2^ over the previous model. Though hippocampal and left frontal lobe measures predicted memory performance, better integrity of the genu of the CC (the proposed route via which right frontal inhibition is effected) was not related to memory scores, partially contradicting the inhibitory hypothesis. Moreover, there was no significant relationship in the entire group between right frontal volumes and memory scores. This provides no support for the hypothesised role of this region in memory scores for the entire group, and runs contrary to the view that right frontal lobe supports retrieval processes during this type of memory test.

**Fig. 2:**
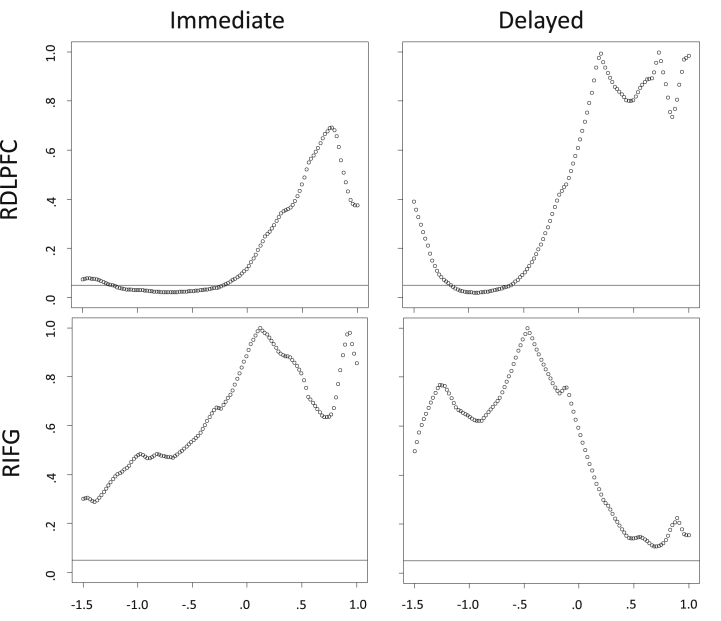
Results of the breakpoint analysis for regressions between right lateral frontal lobe volume controlled for intracranial volume (DLPFC and IFG) and verbal memory score (Immediate and Delayed). Plotted values denote the significance (*y*-axis) of differences between segment slopes across 120 possible breakpoints defined by memory z-score (*x*-axis). Horizontal line denotes *p* = .05. DLPFC: dorsolateral prefrontal cortex, IFG: inferior frontal gyrus.

**Fig. 3:**
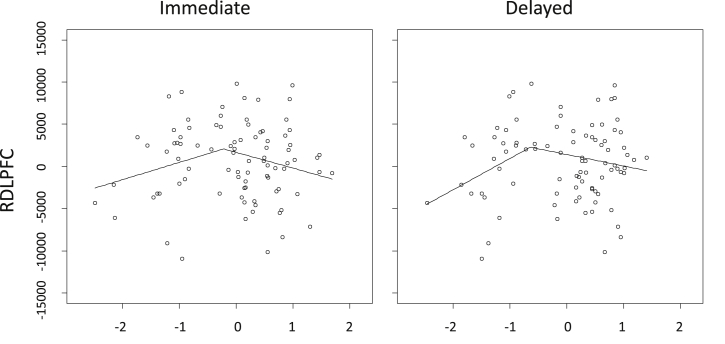
Segmented regression lines plotted for the association between right DLPFC volume controlled for intracranial volume, and verbal memory score (Immediate and Delayed).

Because the breakpoint analyses depend upon a strong scaling assumption, we graphically explored the normality of the distribution and linearity of the relationship either side of the breakpoints, and found these to be acceptable. The results of the breakpoint search algorithm are shown in Fig. 2. It showed that there were a large series of scores for both Immediate and Delayed verbal memory at which the relationship between memory score and right DLPFC were significantly different for performers above and below that point. No breakpoints were identified at which the relationship between either memory score and the right IFG (which lies immediately adjacent to the DLPFC on the lateral convexity of the frontal lobe) were significantly different between segments for high and low performers. To further examine intra-group differences in the predictive value of RDLPFC volumes on memory performance, we selected the significant breakpoint that most evenly distributed power between the two segments. The segmented models were re-parameterized using these breakpoints for Immediate (breakpoint z-score = −.22, *p* = .05) and Delayed (breakpoint z-score = −.64, *p* = .05) verbal memory scores (Fig. 3). For Immediate memory score, higher RDLPFC volumes accounted for 18% of the variance in lower performers (*R*^2^ = .18, *F* (1, 29) = 6.47, *p* = .02), but not for high performers [*R*^2^ = .00, *F* (1, 55) = .23, *p* = .64]. The magnitudes of these associations were significantly different (*z* = 2.23, *p* = .026). For Delayed memory recall, the same relationship was found for low [*R*^2^ = .13, *F* (1, 23) = 3.39, *p* = .08], but not high [*R*^2^ = .00, *F* (1, 61) = .14, *p* = .71] performers. Though the relationship with low performers only showed a trend toward significance, the magnitudes were significantly different (*z* = 2.16, *p* = .031). We then compared the general memory network status (average z-scores of splenium FA, left DLPFC and right hippocampal volume) of those participants either side of the breakpoint. Lower performers had significantly poorer memory network status than higher performers when split by either breakpoint [Immediate: *t* (50.65) = 2.60, *p* *=* .012; Delayed: *t* (32.93) = 2.96, *p* *=* .006]. Group differences in the individual components suggest that this effect is primarily driven by posterior brain differences (Supplementary Table III). These results were generally consistent with the partial compensation hypothesis.

Finally, we investigated whether the exclusion of the 8 left-handed participants had an impact on these results. Left- and right-handers were not significantly different on Immediate or Delayed scores, nor on any of the volumetric or diffusion parameters. Using right-handers only did not significantly alter the magnitude of correlations between MRI variables and memory scores at the group level. Importantly, there was still no association between performance on either memory score and diffusion parameters of the corpus callous genu (*r* range −.04 to .00, *ns*; Supplementary Table IV) and the breakpoint profiles remained significant for right dorsolateral but not right IFG. Moreover, re-parameterizing the models as above was not significantly affected by removing left-handers (Supplementary Fig. I). Higher RDLPFC volumes accounted for 21% of the variance in lower performers [*R*^2^ = .21, *F* (1, 26) = 7.03, *p* = .01], but not for high performers [*R*^2^ = .00, *F* (1, 50) = .19, *p* = .66]. For Delayed memory recall, the same relationship was found for low [*R*^2^ = .11, *F* (1, 21) = 2.47, *p* = .13], but not high [*R*^2^ = .00, *F* (1, 55) = .25, *p* = .62] performers.

## 4 Discussion

We used structural MRI data to test competing hypotheses about memory performance in older people which have arisen from the fMRI literature. On the one hand, right lateral PFC involvement in verbal memory ability might be observed in low performers because right frontal processes are partially compensating for a failing memory network. On the other, it could be indicative of a breakdown of inhibition of the right frontal lobe – one potential route of such inhibition if from the left frontal lobe via the genu of the CC. The data in our study support the account of partial compensation over that of anterior trans-callosal inhibition.

Individual differences in measures of splenium integrity, and right hippocampus and left DLPFC volume each accounted for unique variance in both Immediate and Delayed verbal memory recall scores across the whole group. It is worth noting, however, that unlike the left > right DLPFC and posterior > anterior callosal associations, the hippocampal associations were not significantly lateralised, suggesting a power limitation in the study, rather than implying no role in memory for the left hippocampus. The resultant hierarchical linear regressions are consistent with the idea that these regions form part of a memory network, each component of which contributes uniquely to its overall functioning (Bressler & Menon, 2010; Mesulam, 1990). The finding that left DLPFC volume is related to memory scores is compatible with both hypotheses under examination. However, having better or worse anterior callosal (genu) integrity did not appear to impact on memory performance. This does not readily support the specific inhibitory view under consideration here, whereby poorer memory performance is partially underpinned by reduced inhibition of the right frontal lobe by the left, via the genu of the CC (Buckner & Logan, 2002; Logan et al., 2002; Persson et al., 2006; Sullivan & Pfefferbaum, 2007). Nevertheless, this does not exclude a more general inhibitory account of right frontal activation by any means. It is plausible that right frontal inhibition could originate from another route, or that the inhibitory signal could be weakened by age-related decrements only to the left frontal lobe (from which putative left-to-right inhibitory signals originate), though one would expect poorer callosal integrity to have some bearing on the efficacy with which the inter-hemispheric signal is transmitted. Though not in the anterior portion, we did find that posterior callosal integrity was related to memory performance. The splenium can be considered a component of the hippocampal commissure, comprising cross-hemisphere fibres that connect the hippocampi and tempo–parietal association areas as well as occipital lobes (Knyazeva, 2013). Its integrity has been linked elsewhere to memory functioning and age-related cognitive decline (Hasegawa, 2000; Penke et al., 2010). As such, this work contributes to the extant literature intimating the importance of posterior white matter structures for cognitive ability in older age.

The results of the segmented regression are consistent with the hypothesis that a larger right fronto-lateral area benefits memory performance in older age, but only in those individuals who perform more poorly, and in whom elements of their memory network are failing (de Chastelaine et al., 2011; Rossi et al., 2004). We found that for both Immediate and Delayed memory, the relationship with right frontal volume was 1) significantly positive in lower performers (albeit only a trend for Delayed recall and the right DLPFC), and non-significant in higher performers, and 2) of a significantly different magnitude for low versus high performers. Furthermore, this effect was specific to the dorsal but not inferior portion of the right lateral frontal lobe. Finally, the finding that poorer performers (identified using either Immediate or Delayed breakpoint values) exhibited poorer general memory network status is in line with the suggestion that right frontal involvement in verbal memory performance in poorer performers in older age is driven by a failing memory network. Examination of group differences on individual regions supports the hypothesis that this right frontal involvement is required to supplement change in posterior brain functioning (Davis, Dennis, Daselaar, Fleck, & Cabeza, 2007; Park & Reuter-Lorenz, 2009).

Although the participants in the current study are all generally healthy older adults, who reported no serious neurodegenerative diseases at interview, nor exhibited clinically relevant cerebral features as assessed by a consultant neuroradiologist, it is possible that these performance differences indicate different (and potentially pathological) patterns of ageing; our results indicate that those with poorer splenium integrity exhibited poorer memory performance. Whereas normal healthy ageing is characterised by an anterior greater than posterior decline in callosal FA and a concomitant increase in MD (reviewed in Sullivan & Pfefferbaum, 2007), greater tissue loss in the splenium has been associated with conversion of elderly participants to dementia over a 3-year period when compared to non-converters (overall *n* = 328; Frederiksen et al., 2011). Similarly, an fMRI paradigm involving the immediate (∼7.5 sec) recall of previously-presented numerical stimuli was administered to participants with Alzheimer Disease (*n* = 9) and healthy controls (*n* = 10; Starr et al., 2005). They reported increased superior frontal activation amongst the patient group compared to controls, suggesting that this compensatory activation may be present on a spectrum between normal ageing and Alzheimer Disease. Although our current sample comprises ostensibly normal healthy community-dwelling older adults, changes are thought to occur up to a decade before an eventual diagnosis of probable dementia. It is plausible that poorer performers could be more susceptible to a future conversion to dementia, and prospective data regarding cognitive and neurostructural change over time with the perspective of a pre-morbid baseline will be available to address this question in the future.

### 4.1 Limitations and methodological considerations

Though our participant numbers are not small for an MRI study, they still gave us relatively little power to investigate the complex relationships between estimates of brain structure and verbal memory. Nevertheless, this is a larger study than previously published work on this topic (Duverne et al. 2009: 32 older subjects; de Chastelaine et al. 2011: 36 older subjects). Participants were all male with good tolerance for MRI scanning and motivated to participate in non-incentivised research, and the use of a breakpoint search approach takes account of this skew toward a greater proportion of participants with a higher ability (where an arbitrary median split would not; Fig. 2 indicates a slightly lower proportion of lower performers showing a significantly different effect of higher right DLPFC volume on memory than their high-performing contemporaries). Furthermore, this analysis demonstrates that the reported dissociable involvement of right DLPFC is not merely an artefact of a single arbitrary split, but is present over a large number of possible breakpoints.

It could be argued that our findings are not directly in line with fMRI and lesion studies which indicate a role for the left IFG in verbal memory processes. We found that only left DLPFC and not left IFG volume correlated with our whole-group and high/low verbal memory scores. However, this finding does not suggest that IFG is not involved in these abilities. Rather, correlations between ICV-controlled ROI volumes and cognition broadly represent the degree of change from maximal brain size that is functionally relevant. Examination of the brain variables indicate that the DLPFC volumes showed much wider variance amongst this aged group, consistent with observations that DLPFC structure and function is particularly susceptible to age-related decline (Burzynska et al., 2012; Driscoll et al., 2009; Fjell et al., 2009; Grieve, Clark, Williams, Peduto, & Gordon, 2005; MacPherson, Phillips, & Della Sala, 2002; Raz, Ghisletta, Rodrigue, Kennedy, & Lindenberger, 2010). Thus, although fMRI studies suggest that the IFG is intimately involved in verbal abilities including memory, it is possible that age-related decline in the IFG is less marked than for other frontal regions, and its smaller degree of change is not a primary determinant of individual differences in verbal memory performance in older age.

In spite of suggestions that immediate and delayed memory abilities rely on partially-dissociable neural underpinnings (e.g., Golden, Espe-Pfeifer, & Wachsler-Felder, 2000; Wolk, Dickerson, & Alzheimer's Disease Neuroimaging Initiative, 2011), our data provided little psychometric nor neurostructural evidence to keep these constructs separate. This is in line with the identification of specification errors in the initial factor analysis of the WMS-III (which had previously suggested the separation of immediate and delayed memory) and findings in clinical populations (see Bell, Hermann, & Seidenberg, 2004 for a discussion). Intra-test correlations were higher than those between immediate or delayed measures (Supplementary Table I) and there appeared to be little difference in their relation to the brain variables in question. However, we do note that differences between high and low performers in average memory network integrity appear to be predominantly driven by hippocampal differences for Immediate, but splenium differences for Delayed recall (Supplementary Table III). This could intimate subtle neurobiological distinctions between the two memory constructs, but appropriately powered whole-brain analyses would be required to formally address this question more completely.

Though different neural correlates between high and low performers are apparent in both functional and structural brain MRI, care should be exercised when testing hypotheses and interpreting results in small samples and across methodologies. For example, the BOLD response contrasts reported by Morcom et al. (2003), Duverne et al. (2009) and de Chastelaine et al. (2011) were activation patterns at the time of information presentation for subsequently remembered versus subsequently forgotten items. The present structural MRI data relate to test score only, and so cannot parse apart encoding and retrieval phases – both of which are important for test performance. Thus, we are unable to comment on which phase of memory performance pertains to the neurostructural correlates reported here. Likewise, the absence of fMRI data on the present participants (and lack of structural MRI data in previous fMRI studies) means that one cannot directly assess the correspondence between the functional and structural correlates of verbal memory performance. In particular, it is unclear whether poor performers among our participants would exhibit additional rightward prefrontal BOLD activation when compared to higher performers and young controls. Thus the validity of determining group membership in both this study and previous fMRI research on the basis of performance (rather than functional pattern or neurostructural characteristics) may be suboptimal, though we would predict that low-performers in this study would exhibit stronger right frontal BOLD response than high-performers. Furthermore, our analysis was sensitive to the issue of arbitrarily assigning group membership based on performance alone.

Effort was made to take account of age-related volumetric decline of sub-regions by controlling for ICV, but it is impossible to identify the proportion of individual differences in a particular ROI that are due to accumulated age-related insult, and independent of pre-existing morphological differences in a cross-sectional sample. Ideally, a longitudinal study of structural and cognitive change in progressing old age would be conducted to accurately address this issue. To our knowledge, no such longitudinal studies have explicitly addressed the question of verbal memory performance-based differences in frontal hemispheric laterality in older age thus far. Moreover, volumetric measures cannot account for age-related changes in receptor density and distribution which may also change with increasing age (Park & Reuter-Lorenz, 2009). Measures of non-fronto-cortical regions, sub-cortical structures, other major tracts such as the fornix (implicated in hippocampal-PFC connectivity; Metzler-Baddeley, Jones, Belaroussi, Aggleton, & O'Sullivan, 2011) are absent, but would allow a fuller account of structure-function relationships.

Finally, no self-report was taken regarding participants' encoding strategies. Differences in verbal memory strategy have previously been reported to elicit different functional activation patterns, but age-related over-recruitment in the right hemisphere itself appeared resistant to training in a more successful verbal encoding strategy (Logan et al., 2002). It could therefore be possible that performance was dictated by strategy adoption, but whether strategy adoption in older age is constrained by brain integrity or vice versa is an interesting question that could be addressed in future work.

### 4.2 Conclusions

In summary, the current study provides a novel perspective on two competing theories that have arisen from the fMRI literature, using neurostructural data (structural and diffusion MRI). We found little evidence supportive of the hypothesis that poorer performers exhibit a breakdown in cross-hemisphere inhibition of the right PFC by the left PFC via the genu of CC. Instead, we identified divergent neural correlates for verbal memory recall between high and low performers in older age, indicative of a partially compensatory role of the right DLPFC among individuals who are performing more poorly, possibly to supplement changes in posterior and left fronto-lateral functioning (Davis et al., 2007; Park & Reuter-Lorenz, 2009). Future studies aiming to improve our understanding of this aspect of brain ageing and its cognitive sequelae will ideally increase participant numbers and combine structural, diffusion and fMRI modalities with an examination of strategy adoption and a wider view of other brain regions that may contribute to verbal memory ability.

## Funding

This research and LBC1936 phenotype collection were supported by Age UK (The Disconnected Mind project). It was undertaken in the Centre for Cognitive Ageing and Cognitive Epidemiology (http://www.ccace.ed.ac.uk)—part of the cross council Lifelong Health and Wellbeing Initiative—which is supported by funding from the UK's Biotechnology and Biological Sciences Research Council, the Economic and Social Research Council and the Medical Research Council (MR/K026992/1). Brain imaging took place in the University of Edinburgh in the Brain Research Imaging Centre (http://www.bric.ed.ac.uk) which is part of the SINAPSE collaboration (http://sinapse.ac.uk).

## Figures and Tables

**Table 1:** Descriptive statistics of study variables.

	*n*	Units	Mean (SD)	Max
Age	90	years	73.10 (.40)	
MMSE	90		28.54 (1.51)	30
HADS total	90		6.71 (4.39)	32
Logical Memory I	90		44.37 (11.43)	75
Logical Memory II	90		27.62 (8.82)	50
VPA I	89		19.88 (7.76)	32
VPA II	89		6.09 (2.26)	8
ICV	90	mm^3^	1,545,481 (113,685)	
L Hippocampus	89	mm^3^	3079 (392)	
R Hippocampus	89	mm^3^	3376 (412)	
L DLPFC	88	mm^3^	25,064 (5644)	
R DLPFC	88	mm^3^	24,477 (5410)	
L IFG	88	mm^3^	16,238 (2946)	
R IFG	88	mm^3^	15,715 (3110)	
Genu FA	85		.40 (.05)	1
Genu MD	85	×10^−6^ mm^2^/sec	789.18 (79.14)	
Splenium FA	88		.49 (.07)	1
Splenium MD	88	×10^−6^ mm^2^/sec	979.63 (183.73)	

MMSE: mini-mental state exam; ICV: intracranial volume; L: left; R: right; DLPFC: dorsolateral prefrontal cortex, IFG: inferior frontal gyrus; FA: fractional anisotropy; MD: mean diffusivity.

**Table 2:** Hierarchical linear regression showing the increase in verbal memory score variance accounted for by brain imaging variables.

Covariates (Immediate Recall)	*β1*	*β2*	*β3*	*F*	*df*	*R*^*2*^	*p*
+Splenium_FA	.28			7.20	1, 84	.08	.009
+Splenium_FA + Right Hippocampus	.25	.29		6.37	2, 82	.13	.003
+Splenium_FA + Right Hippocampus + Left DLPFC	.25	.28	.11	5.16	3, 79	.16	.003

Covariates (Delayed Recall)	*β1*	*β2*	*β3*	*F*	*df*	*R*^*2*^	*p*
+Splenium_FA	.32			7.56	1, 84	.10	.003
+Splenium_FA + Right Hippocampus	.29	.27		7.01	2, 82	.15	.001
+Splenium_FA + Right Hippocampus + Left DLPFC	.30	.26	.15	6.22	3, 79	.19	.0008

FA: fractional anisotropy; DLPFC: dorsolateral prefrontal cortex. Hippocampal and DLPFC are volumes (mm^3^) controlled for intracranial volume.
