# Supplementary material for: Compensation or inhibitory failure? Testing hypotheses of age-related right frontal lobe involvement in verbal memory ability using structural and diffusion MRI
Source: Cortex. 2015 Feb;63:4–15. doi: 10.1016/j.cortex.2014.08.001 (PMC4317301; doi:10.1016/j.cortex.2014.08.001)
Supplement: Supplementary file 1 [file mmc1.docx]

**Supplementary Material**

*Supplementary Table I.* Correlations among verbal memory scores.

|  | Logical Memory | | VPA | |
| --- | --- | --- | --- | --- |
|  | I | II | I | II |
| Logical Memory I | - |  |  |  |
| Logical Memory II | **.89***** | - |  |  |
| VPA I | **.56***** | **.57***** | - |  |
| VPA II | **.48***** | **.50***** | **.88***** | - |

VPA: verbal paired associates test. ******p* < .0001.

*Supplementary Table II.* Correlations between frontal lobe regional volumes (ICV-adjusted) and indices of callosal integrity.

|  | DLPFC | | IFG | | GENU | | SPLENIUM | |
| --- | --- | --- | --- | --- | --- | --- | --- | --- |
|  | Left | Right | Left | Right | FA | MD | FA | MD |
| DLPFC Left | - |  |  |  |  |  |  |  |
| DLPFC Right | **.50***** | - |  |  |  |  |  |  |
| IFG Left | **.26*** | **.31**** | - |  |  |  |  |  |
| IFG Right | .13 | **.36**** | **.56***** | - |  |  |  |  |
| Genu FA | .15 | .05 | .17 | -.05 | - |  |  |  |
| Genu MD | -.11 | -.08 | -.07 | .05 | **-.61***** | - |  |  |
| Splenium FA | .10 | -.00 | .13 | .12 | .12 | .06 | - |  |
| Splenium MD | -.11 | -.10 | -.09 | -.15 | .01 | .04 | **-.83***** | - |

DLPFC: dorsolateral prefrontal cortex; IFG: inferior frontal gyrus; FA: fractional anisotropy; MD: mean diffusivity. ***p* < .01, ******p* < .001.

*Supplementary Table III.* Group differences in memory network component indices.

|  | HC | | Left DL | | Splenium FA | |
| --- | --- | --- | --- | --- | --- | --- |
|  | *t* | *p* | *t* | *p* | *t* | *p* |
| Immediate Recall | 2.22 | .03 | 0.93 | .35 | 1.39 | .17 |
| Delayed Recall | 1.68 | .10 | 1.14 | .26 | 2.72 | .01 |

Groups are split at the memory score identified from the breakpoint analysis for Immediate and Delayed verbal memory recall. HC: hippocampus; DL: dorsolateral; FA: fractional anisotropy; HC and DL are volumes (mm^3^) controlled for intracranial volume.

*Supplementary Table IV.* Correlations between MRI variables and verbal memory performance at the group level, for right-handers only.

|  | HC | | DL | | IFG | | | Genu | | Splenium | |
| --- | --- | --- | --- | --- | --- | --- | --- | --- | --- | --- | --- |
| **Mean Z-Score** | Left | Right | Left | Right | Left | Right | | FA | MD | FA | MD |
| Immediate Recall | .15 | **.29**** | **.23*** | .06 | .04 | | .12 | -.03 | -.04 | **.29**** | -.22^†^ |
| Delayed Recall | .18 | **.27*** | **.26*** | .11 | .11 | | .14 | .00 | -.02 | **.31**** | **-.23*** |

HC: hippocampus; DL: dorsolateral; IFG: inferior frontal gyrus; FA: fractional anisotropy; MD: mean diffusivity. HC and DL are volumes (mm^3^) controlled for intracranial volume.

*Supplementary Figure I.* Results of the breakpoint analysis for regressions between right dorsolateral (top row) and right inferior frontal gyrus (bottom row) and verbal memory score (Immediate and Delayed) for right-handers only. Plotted values denote the significance (y-axis) of differences between segment slopes across 120 possible breakpoints defined by memory z-score (x-axis). Horizontal line denotes *p* = .05.
